# Efficient organic photomemory with photography-ready programming speed

**DOI:** 10.1038/srep30536

**Published:** 2016-07-26

**Authors:** Mincheol Kim, Hyejeong Seong, Seungwon Lee, Hyukyun Kwon, Sung Gap Im, Hanul Moon, Seunghyup Yoo

**Affiliations:** 1 KAIST, School of Electrical Engineering, Daejeon, 34141, Korea; 2 KAIST, Department of Chemical and Biomolecular Engineering, Daejeon, 34141, Korea

## Abstract

We propose a device architecture for a transistor-type organic photomemory that can be programmed fast enough for use in electrical photography. Following the strategies used in a flash memory where an isolated charge storage node or floating gate is employed, the proposed organic photomemory adopts an isolated photo-absorption zone that is embedded between upper and lower insulator layers without directly interfacing with a semiconductor channel layer. This isolated photo-absorption zone then allows the device to operate in electrically ‘on’ state, in which the high electric-field region can have a maximal spatial overlap with the illuminated area for efficient and facile light-programming. With the proposed approach, a significant threshold voltage shift is attained even with the exposure time as short as 5 ms. High quality dielectric layers prepared by initiated chemical vapor deposition ensure erasing to occur only with electrical signal in a controlled manner. Retention time up to 700 s is demonstrated.

Recent advances in organic electronics have proven their significance and competence in optoelectronic applications^1^,^2^,^3^,^4^,^5^,^6^. In particular, organic photosensors have been regarded promising due to their numerous potential applications in image sensing^7^,^8^,^9^, optoelectronic communication^3^,^4^,^5^,^6^, and health care monitoring^10^,^11^,^12^,^13^. They have been developed with various structures and operating mechanisms^14^ such as photoconductors^15^,^16^, photodiodes^17^,^18^,^19^, phototransistors^20^,^21^,^22^, and photomemories. Among them, photomemories combine both photosensors and memories, and thus enable simplified circuitry by eliminating the need for independent memory components to store the output of the photosensors^23^,^24^,^25^,^26^,^27^,^28^,^29^. In particular, photomemories based on a transistor structure provide additional advantages, in that they allow for further simplification of pixel-level structure by serving as photosensors, memories, and switching devices all at once. For that reason, organic photomemories based on a transistor structure have been proposed with various operating mechanisms, including a photochromic channel semiconductor^23^, a quantum dot embedded channel semiconductor^25^, interface trap^24^,^26^,^28^, or an additional layer inserted between a channel semiconductor and a gate dielectric layer^27^. (Hereafter, “organic photomemory (OPM)” refers to the devices based on a transistor structure, unless specified otherwise.)

Most OPMs reported to date, however, have needed prolonged light-programming time (*t*_prgm_), typically longer than a second. To the best of our knowledge, even the fastest device required a *t*_prgm_ as large as 0.25 s^27^. A short *t*_prgm_ is an important performance factor for any practical applications of photosensing or imaging systems. For example, in electrophotography, minimum shutter speed is ultimately limited by the response time of the photosensor. In an X-ray imaging system, the necessary amount of X-ray exposure may be too large for a human body if a sensor does not respond fast enough. Hence, substantial progress needs to be made to reduce *t*_prgm_ as much as possible. Here, we propose a novel device architecture for OPMs based on an isolated photo-absorption zone (PAZ) that leads to a significantly reduced *t*_prgm_.

## Results and Discussion

**Figure 1:**
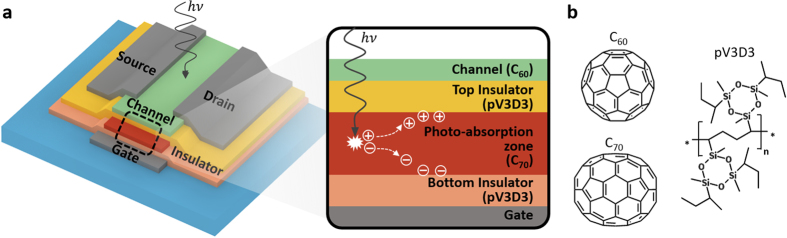
An overview of the proposed organic photomemory device. (**a**) The structure of the proposed organic photomemory (OPM) device with isolated photo-absorption zone (PAZ), an enlarged cross-sectional structure to depict exciton generation, charge separation, and charge trapping processes by light-programming. (**b**) Molecular structures used in the device.

The structure of the proposed OPM device is presented in Fig. 1a. It is based on a bottom-gate, top-contact n-channel organic thin-film transistor (OTFT), adopting a semiconductor channel made of evaporated C_60_. It incorporates a 30 nm-thick C_70_ layer as a PAZ in between two polymer gate insulating layers, poly(1,3,5-trimethyl-1,3,5-trivinyl cyclotrisiloxane) (pV3D3) deposited by initiated chemical vapor deposition (iCVD), which has recently been demonstrated as a versatile gate insulator that can be down-scaled to a few tens of nanometers while retaining excellent insulating properties (Fig. 1b)^30^.

The overall device structure in this work is inherited from a flash memory, which is in essence a transistor with a floating gate embedded in between a tunneling dielectric layer and a blocking dielectric layer^31^. For a photo-induced memory programming, the floating gate is replaced with a PAZ. In this way, the PAZ is isolated from the channel itself, and from the interface made with the channel, leading to an inherent advantage in that it allows one to individually choose the material best suited for the photosensitive function and the semiconductor role, respectively, with little concern for cross-interference. Unlike a typical flash memory where a tunneling dielectric layer is thinner than a blocking dielectric layer, the thicknesses of the two insulating layers are designed to be the same, so that the device is programmed not by carriers exchanged through a tunneling dielectric layer by tunneling, but by the carriers photo-generated within the PAZ.

The principle of operation behind the light-induced programming in the proposed device is schematically shown in the enlarged cross-sectional view in Fig. 1a, and involves several sequential steps: 1) generation of excitons in the PAZ upon the absorption of light; 2) exciton dissociation into separate electrons and holes under the influence of the gate-bias (*V*_G_) induced electric field; 3) drift of the separated electrons and holes toward the opposite sides of the PAZ/dielectric interfaces; and 4) accumulation/trap of electrons and holes at the PAZ/bottom- and top-insulator interfaces, respectively. This spatially separated distribution of positive and negative charges causes an internal potential difference across the PAZ and induces a threshold voltage shift (*ΔV*_th_) in the opposite direction with respect to the applied *V*_G_. The relationship between *ΔV*_th_, and the photo-generated and trapped carrier density (*n*_charge_; in cm^−2^) is described by^32^,^33^:


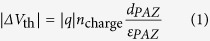


where *q* is the electronic charge, *d*_PAZ_ and *ε*_PAZ_ is the thickness and the permittivity of a given PAZ, respectively.

**Figure 2:**
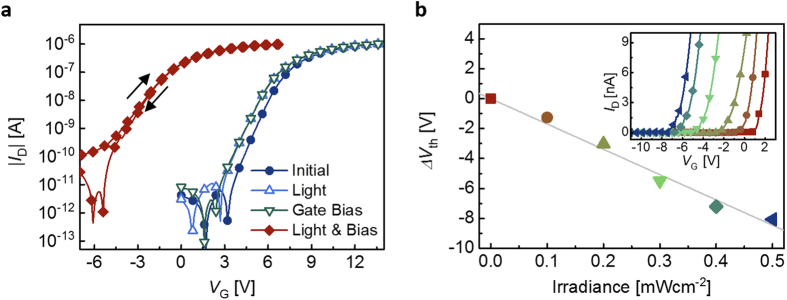
Light-programming effect on the transfer characteristics of the proposed OPM. (**a**) Transfer curves of the photomemory in the linear region with *V*_D_ = 1 V under various stimulus conditions, in which “Light” and “Bias” correspond to a white LED light of 3.76 mWcm^−2^ and a gate bias pulse of 22 V in 50 ms, respectively. (**b**) Irradiance response of the photomemory with a collimated green LED (*λ* = 528 nm) as a light source and gate bias of 22 V in 50 ms and the gray line is its linear regression (inset: linear region transfer curves corresponding to the each measurement points of the irradiance response test).

Figure 2a presents the transfer curves of the proposed OPM device at the initial state and after various stimulus conditions, which consist of illumination (3.76 mWcm^−2^; white LED) and/or *V*_G_ pulse (22 V, 50 ms) with both of the source and drain electrodes grounded. The electrical characteristics of the device, except *V*_th_, remain virtually unaltered after light-programming. A significant *ΔV*_th_ in a negative direction is observed when both the light and the *V*_G_ pulse are applied simultaneously, while only a negligible shift is observed when the light and *V*_G_ pulse stimuli are applied individually. (Hereafter, “light-programming” thus refers to the case where both illumination and *V*_G_ pulse stimuli are applied at the same time, unless specified otherwise.)

Note that the light-programming voltage of 22 V is larger than ca. 12 V, typically used for programming of non-volatile flash memories. To cope with this issue to some degree, one may scale down the bottom and top dielectric layers. While it is challenging to maintain the insulating properties of polymeric layers at thickness below several tens of nanometers, iCVD-based pV3D3 layers were shown to hold well their excellent insulating characteristics even at a thickness of 10–20 nm or less^30^, making it likely to further bring down the programming bias by decreasing the thickness of the bottom and top pV3D3 layers.

The role of the PAZ in light-programming is verified using a device having essentially the same structure but without the PAZ, which does not exhibit any sign of light-programming function (See Supplementary Fig. S1). This is in a clear contrast with the proposed OPM, which exhibits a linear increase in |*ΔV*_th_| as the irradiance of the incident light increases (Fig. 2b).

From the discussions made above regarding the operation mechanism and the experimental photo-electrical characteristics shown in Fig. 2, it can be easily seen that the PAZ is the most important component affecting the high photo-responsivity of the proposed OPM, and C_70_ was adopted due to its high photon absorption in the visible spectral range (Supplementary Fig. S2). Depending on applications, C_70_ may be replaced with another highly absorbing organic or inorganic semiconductor provided that process for its preparation is compatible with the overall fabrication process. If desired, materials with a particular spectral absorption band may also be chosen, for example, for a targeted color sensitivity.

**Figure 3:**
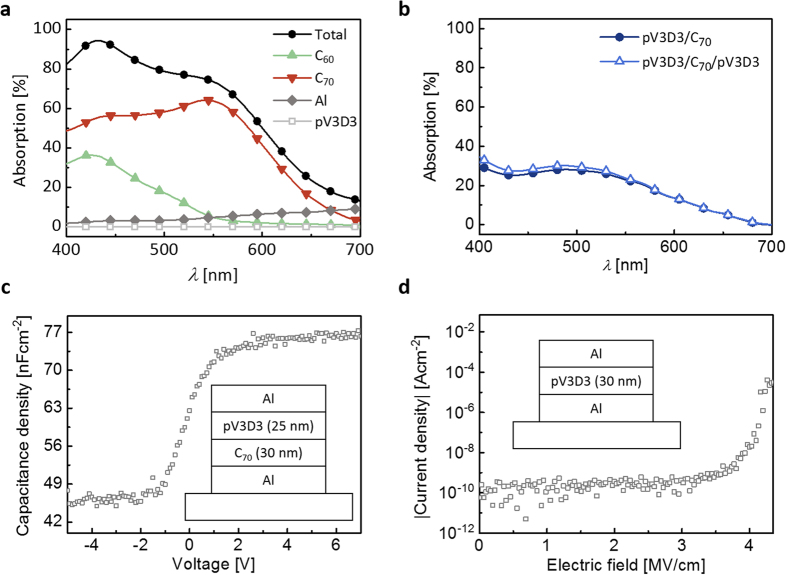
The properties of key components in the proposed OPMs and their process compatibility. (**a**) Calculated absorptions of the C_70_ and C_60_ layers in the channel region of the proposed device, Al/pV3D3/C_70_/pV3D3/C_60_, based on thin-film optics (see Materials and Methods section for details). (**b**) Measured absorptions of the C_70_ layer before and after pV3D3 deposition onto it. (**c**) Capacitance density vs. applied voltage characteristics of an Al/C_70_/pV3D3/Al device with the thickness of C_70_ and pV3D3 as 30 nm and 25 nm, respectively. (**d**) Leakage current density vs. applied electric field characteristics of an Al/pV3D3/Al device with a 30 nm-thick pV3D3 layer.

For the channel semiconductor, high electrical performance and low photo-absorption are preferred. C_60_ was previously shown to exhibit high mobility of up to 1.5 cm^2^V^−1^s^−1^ and stable operation to electrical stress when deposited on the pV3D3 surface in a TFT geometry (Fig. 2a; Supplementary Fig. S3). The photo-absorption of C_60_ is much smaller than that of C_70_ due to its spherical symmetry (Fig. 3a)^34^, and thus a reduction in the photo-absorption in the C_70_ PAZ due to the optical “filter effect” of C_60_ can be minimized (Fig. 3a; Supplementary Fig. S2). The observed lack of light-programming behavior in the control device without the PAZ suggests that the C_60_ channel layer is unable to show any significant photo-induced memory by itself, and the absorption in the C_70_ layer is indeed a main origin for the photo-response (Fig. 3a; Supplementary Fig. S1).

For the C_70_ layer to properly operate as a PAZ, its electrical and optical characteristics should not be degraded during the device fabrication process. This could be critical because organic semiconductors are typically prone to damage by heat or reactive chemicals which might be introduced in the process of depositing the insulating layer on its top. In this respect, the iCVD processed pV3D3 is expected to play a key role, not only for its excellent insulating properties but also for the damage-free benefit of the iCVD process, which is a solvent-free, vapor-based technique performed at a low substrate temperature of approximately 40 °C 30. Absorption spectra of a C_70_ layer shown in Fig. 3b turn out to be virtually identical before and after the deposition of pV3D3. Similar immunity of C_70_ to the pV3D3 deposition process is also observed in electrical properties, as well in the capacitance-voltage characteristics obtained from the Al/C_70_/pV3D3/Al device (Fig. 3c); the adequate switching behavior from charge depletion to accumulation upon increase in bias can be clearly seen, as expected. All these results indicate that C_70_ retains its semiconductor characteristics from both optical and electrical perspectives.

It is also noteworthy that the two insulating layers play a dual role; they work as gate dielectrics and, at the same time, provide the PAZ with physical and electrical isolation from the gate and the channel. Approximately 38 nm-thick pV3D3 polymer layers are used for both of the insulating layers. They show a leakage current density of around 10^−9^ Acm^−2^ for an electrical field intensity of up to 3 MVcm^−1^ (Fig. 3d)^30^. This excellent insulating property provides sufficient electrical isolation of the PAZ during the light-programming and reading processes, so that the light-induced charges will not be interfered by exchange of charges with the gate or channel via tunneling. A sudden increase in the leakage current density is observed with an electric field over 3.5 MVcm^−1^, and it was previously shown to originate from Fowler-Nordheim tunneling (see Supplementary Fig. S4 for details), indicating that the ideal insulating behavior of pV3D3 layers is enabled by their sufficiently low defect densities^30^. In terms of optical property, the pV3D3 layers do not absorb any visible light due to its wide bandgap over 8 eV (Fig. 3a, Supplementary Figs S2 and S5)^30^, and that is also beneficial for the proposed photomemory operation originating in the absorption of PAZ.

**Figure 4:**
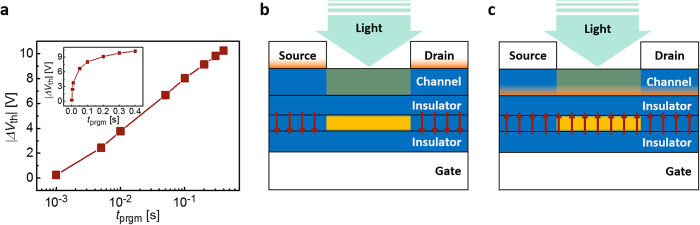
Light-programming speed of the proposed OPM–its structural advantage. (**a**) Programming speed of the photomemory device with 22 V of gate bias and a white LED as a light source (3.76 mWcm^−2^). Schematic illustrations of light path and electric field distribution (**b**) in turn-off state with the depleted channel, and (**c**) in turn-on state with the accumulated channel. The yellow region is where most light absorption occurs, the orange region is where charges are accumulated by *V*_G,_ and the arrows denote electric field across the PAZ.

*ΔV*_th_ was measured as a function of *t*_prgm_ using the collimated illumination (3.76 mWcm^−2^) from a white LED and a gate bias of 22 V (Fig. 4a). Most of all, it is noteworthy that the device exhibits a distinguishable *ΔV*_th_ over 2.5 V even with the gate bias pulse as short as 5 ms. This level of programming time is far smaller than those of the other OPM reported so far. In most of those devices, light-programming was performed in the electrically ‘off’ state because their photosensitive parts involved channels themselves; in that case the gate-induced charges in the ‘on’ state could easily shadow the photo-induced effect and thus ‘off’ state programming is preferred. However, the gate-induced electric field in the ‘off’ state is applied mostly under the area of the source/drain electrodes that is overlapped with a gate electrode. This could be disadvantageous because light enters into the device layers (channels or dielectrics) mostly through the channel area that is not covered by the metallic source/drain electrodes (Fig. 4b)^23^,^24^,^25^,^26^,^27^,^28^,^29^. In that case, the light-programming, requiring both electric field and photon-absorption, is discouraged by significantly low electric field in the channel area, or by the lack of photons in the region under source/drain electrodes.

In contrast, the OPM proposed in this work conducts light-programming in the ‘on’ state thanks to the isolation of the PAZ from its channel (Fig. 1a). In this way, the electric field by *V*_G_ can be applied to the whole channel area using accumulated charges at the channel; consequently, locations where both electric field and light are strong can be well matched with a maximal spatial overlap, enabling fast and efficient photomemory operation (Fig. 4c). The external quantum efficiency (*η*_EQE_) of the proposed OPM can be estimated for constant, uniform illumination of a monochromatic light with the wavelength of *λ* by:


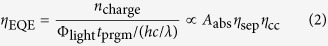


in which Φ_light_ is the irradiance of the incident light, *h* is the Planck constant, *c* is the speed of light, *A*_abs_ is the absorption at the photoactive layer (PAZ in the present work), *η*_sep_ is the probability of the charge separation from excitons, and *η*_cc_ is the efficiency for photo-generated carriers to be collected, i.e., transported and trapped, at the PAZ/dielectric interfaces. Note that the positive correlation of *η*_EQE_ on the magnitude of electric field mainly results from those of *η*_sep_ and *η*_cc_^35^,^36^. Hence, low electric field across PAZ would result in low EQE and would thus require a long *t*_prgm_ for *n*_charge_ to reach a value corresponding to a target *ΔV*_th_.

In the case of the proposed OPM used in Fig. 2b, *η*_EQE_ is estimated to be as large as ca. 10% at *λ* = 528 nm for *t*_prgm_ = 50 ms, being consistent with the observed efficient, fast programming capability of the proposed OPM. It is noteworthy that *η*_EQE_ is a monotonically decreasing function of *t*_prgm_ because *n*_charge_ is a sublinear function of *t*_prgm_, as can be seen from Fig. 4a, where *ΔV*_th_ (and thus *n*_charge_) is proportional to the logarithm of *t*_prgm_. This saturation like-behavior originates from the fact that *η*_sep_ and *η*_cc_ decrease as the electric field decreases^35^,^36^ and that the electric field in the PAZ due to *V*_G_ is reduced by the compensating field resulting from accumulated/ trapped charges at each interface of PAZ that increase with *t*_prgm_.

The response of the proposed OPM may also be described in terms of the exposure (*H*) used for photographic films^37^, which is a parameter defined as the irradiance of light times the exposed time. This is particularly the case because the signal in the proposed OPM depends on *n*_charge_ formed during a given period of time rather than *n*_charge_ per unit time. As the optical density is given by a monotonically increasing function of *H* in a photographic film, |*ΔV*_th_| increases with *H* in the proposed OPMs (Supplementary Fig. S6) with *H* of the OPM defined by:


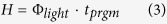


Eventually, the detectivity of a given OPM may be determined by the minimum *H* leading to a measurable |*ΔV*_th_| considering its error range. The proposed OPM exhibited |*ΔV*_th_| as large as a few volts even with the *H* of 0.015 mW·s·cm^−2^, confirming its effectiveness in utilizing the incident light, leading to efficient and fast programming.

**Figure 5:**
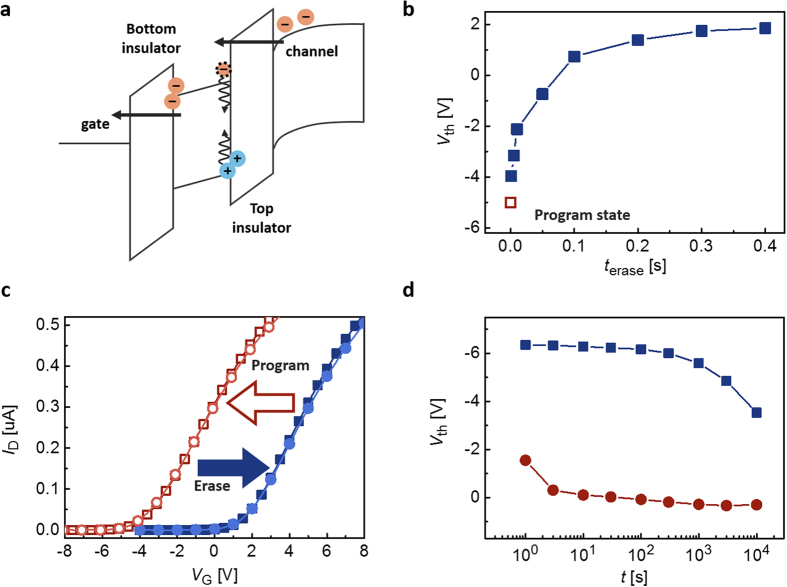
Photomemory characteristics of the proposed OPM. (**a**) Schematic of energy band diagram for electrical erasing of the photomemory device. (**b**) Speed of the electrical erasing of the photomemory device in programmed state. (**c**) Transfer curves of repeatedly programmed and erased state in the following order: 1. erased (solid square) 2. programmed (open square) 3. erased (solid circle) 4. programmed (open circle). (**d**) Retention characteristics of the photomemory device (square: programmed state, circle: erased state).

To erase the stored information in the proposed OPM, one approach would be to apply gate bias with the opposite polarity to that of programming operation. However, this is expected to be inefficient for the same reason that the effective area of the gate-induced electric field is limited, in the turned-off state, only to the region under the source/drain electrodes while the charges to be erased are distributed in the region under the channel. Hence, we propose an erasing signal having the same polarity as the *V*_G_ for programming. If one applies *V*_G_ large enough, Fowler-Nordheim tunneling current occurs through both bottom and top insulating layers, and thus trapped carriers at the bottom or top C_70_/pV3D3 interfaces can be taken out or recombined with injected carriers with the opposite polarity (Fig. 5a). The erasing *V*_G_ in this scheme is estimated to be 40 V, which corresponds to an electric field in the pV3D3 layers of over 4.4 MVcm^−1^, corresponding to the Fowler-Nordheim tunneling regime in the leakage current density vs. electric field intensity characteristics shown in Fig. 3d (see Supplementary Fig. S7 for further details). For the programmed device with *ΔV*_th_ of −6 V, the negatively shifted *V*_th_ is shown to be fully recovered with this erasing *V*_G_ and saturated at the initial *V*_th_ after about 200 ms, indicating that stored carriers are completely bleached (Fig. 5b). The energy level alignment between Al gate electrode and C_70_, C_60_ semiconductor layers and the identical thickness of the bottom and top pV3D3 layers ensures the same tunneling current through both the bottom and top insulating layers, thereby preserving the charge neutrality during the erase process^38^,^39^,^40^. The charge neutral behavior in the PAZ can be verified by the fact that memory characteristics remain almost constant with the repeated programming and erasing cycles (Fig. 5c). The proposed photomemory exhibited homogeneous light-programming and electrical erasing properties over 50 times of endurance test (Supplementary Fig. S8).

The retention time for the programmed state was about 700 s and 10 ks, to maintain 90% and 50% of the programmed *ΔV*_th_, respectively (Fig. 5d). 700-s retention will be long enough for light or image sensor applications in which brightness can be mapped in space at a given time and refreshed in a short interval. With such memory characteristics, “rolling-shutter” operation typically needed for CMOS-based image sensors is not necessary, and instead desirable “global shutter” operation can be realized^41^. If one is simply to use the criterion to verify the presence of light, 10 ks retention may suffice for various short-term applications where the stored visual information may be retrieved within several hours.

In summary, an organic photomemory (OPM) with an isolated photo-absorption zone (PAZ) was proposed in a flash memory-like transistor structure. The PAZ made of C_70_ was surrounded by iCVD-grown top and bottom insulator layers and thus isolated from the channel. This isolation of the PAZ allows light-programming to be done with the channel ‘on,’ enabling full use of the PAZ under the whole channel region and thus leading to substantial reduction in light-programming time, down to as low as 5 ms. This result carries significant meaning as it is the first report of an OPM whose light-programming speed is comparable to the ordinary frame speed of a camera. Tunneling-based electrical erasing of the photo-stored information was also performed, demonstrating that the OPM could be used repeatedly. Together with the extensibility to individual color sensing or global shutter system as well as the simplicity that can be offered, the proposed OPM may open up the possibility for next-generation applications such as image- or X-ray mappers that would benefit from the large-area capability and form factor advantages of organic materials.

## Methods

### Device fabrication

Glass substrates were cleaned in an ultrasonic bath with detergent-dissolved deionized water, deionized water, acetone, and 2-propanol in sequence, and were then dried in a vacuum oven. Al (70 nm) was thermally evaporated in a vacuum chamber for the gate electrode. pV3D3 was deposited by iCVD process as a bottom insulating layer, and then, a C_70_ film was evaporated as a PAZ in the vacuum chamber, followed by the deposition of the top pV3D3 layer by iCVD process^30^. Finally, C_60_ (50 nm) and Al (70 nm) were deposited by thermal evaporation for a semiconducting channel layer and source/drain electrodes. Sample transfer process was controlled such that there should be no ambient air exposure after C_70_ deposition throughout the fabrication and characterization process. Every layer except the insulating layers was patterned by metal shadow masks.

The device used in the irradiance-dependence measurement had the thicknesses of 59 nm, 26 nm, and 55 nm for top insulator, PAZ, and bottom insulator, respectively, and the rest of the results were obtained from a device having the thicknesses of 36 nm, 30 nm, 39 nm for the respective layers. The thickness of the pV3D3 layers and the C_70_ layer was estimated from their capacitance in a metal-insulator-metal structure with Al for both metal electrodes. All the memory devices had a channel length and width of 200 μm and 1000 μm, respectively.

### Optical analysis

The calculation of absorptions was performed using a MATLAB code based on a transfer matrix formalism dealing with the optical properties of a multi-layer thin film structure^42^. The optical constants of each layer composing the proposed device were obtained by spectroscopic ellipsometry (Supplementary Fig. S2). Absorption spectra were measured in ambient air using a UV/Vis/NIR Spectrometer (Lambda 950, Perkin Elmer).

### Device characterization

Devices characteristics were measured in a nitrogen-filled glove box under controlled dark ambient. Both green and white LED light sources were collimated and had the area of illumination large enough to cover the whole channel region of the measured devices. The dark condition was maintained during the characterization of the OPM devices.

The capacitances were measured using a precision LCR meter (HP4284, Agilent) and the current versus voltage characteristics were measured using a semiconductor parameter analyzer (HP4155A, Agilent). The field effect mobility was extracted by a transconductance method from linear region transfer characteristics^43^.

## Additional Information

**How to cite this article**: Kim, M. *et al*. Efficient organic photomemory with photography-ready programming speed. *Sci. Rep*. **6**, 30536; doi: 10.1038/srep30536 (2016).

## Supplementary Material

Supplementary Information
