# Supplementary material for: Efficient organic photomemory with photography-ready programming speed
Source: Sci Rep. 2016 Jul 26;6:30536. doi: 10.1038/srep30536 (PMC4960596; doi:10.1038/srep30536)
Supplement: Supplementary Information [file srep30536-s1.pdf]

# Efficient organic photomemory with photography-ready programming speed

*Mincheol Kim, Hyejeong Seong, Seungwon Lee, Hyukyun Kwon, Sung Gap Im, Hanul Moon, and Seunghyup Yoo**

## Supplementary information

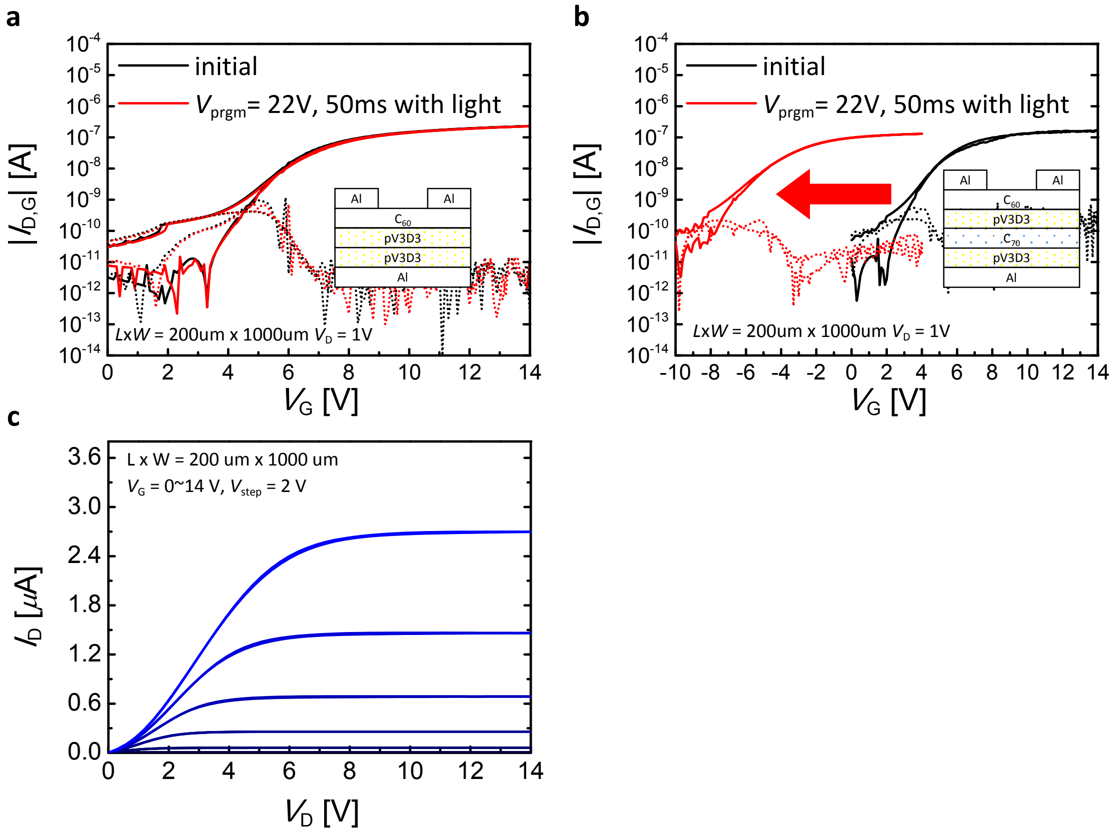


**Supplementary Figure S1. Transfer characteristics of devices with or without photo-absorption zone and the device output characteristic.** a) Control device without $C_{70}$ as photo-absorption zone (PAZ) and b) the device with PAZ. c) is an output curve of the photomemory at the initial state.

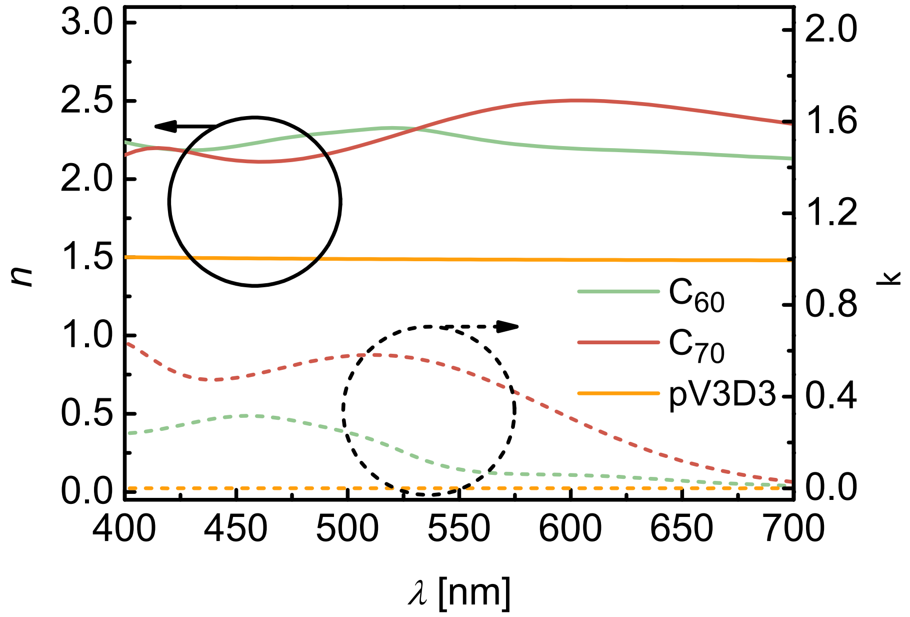


**Supplementary Figure S2. Optical constants of the materials.** Optical constants of the materials used in the proposed organic photomemory measured by ellipsometry for the calculation of absorption shown in Figure 2a.

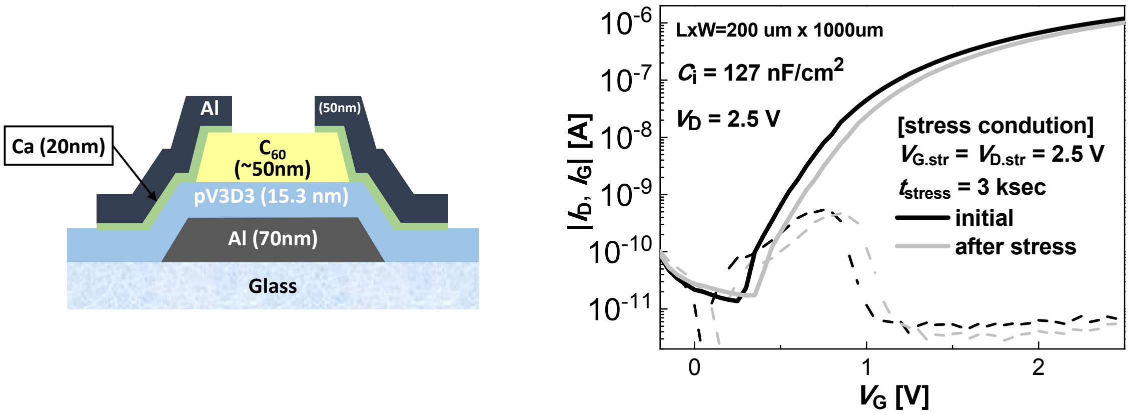


**Supplementary Figure S3. Electrical stability of $C_{60}$ transistor based on pV3D3 gate dielectric.** Device structure and electrical stability of $C_{60}$ transistor with pV3D3 as a gate insulator. Constant voltage stress test showed only 0.15V of $V_{th}$-shift after 3000 s of applied bias condition One may wonder if the observed $V_{th}$ shift with both light and bias applied in the proposed photo-memory is mainly due to the photo-induced effect or simply from the gate-bias stress effect. One may be assured that it is indeed the former, as designed, for the following reasons: (i) $V_{th}$ shift due to the bias stress is toward positive direction, which is opposite to the direction of $V_{th}$ shift resulting from the light-programming response; and (ii) the number of charge density at the interface between a channel and a dielectric layer and the field across the overall dielectric layers, which are key factors for bias-stress effect,[1] are 1.5 times larger for the stress condition used for the reference device than those used for photomemory devices in the experiment investigating light-programming effect (See Fig. 2a in the main text). In addition, the lack of $V_{th}$ shift observed with only the pulsed gate bias applied (Fig. 2a in the main text) also indicates that such a pulsed bias condition is not sufficient to cause severe bias stress effect, considering that light programming involves a pulsed bias with the same duration and magnitude. From all of the things mentioned above, it is likely that $V_{th}$ shift due to the bias stress, if any, would not undermine our conclusion that $V_{th}$ shift results mainly from the light-programming response in the proposed organic photomemory.

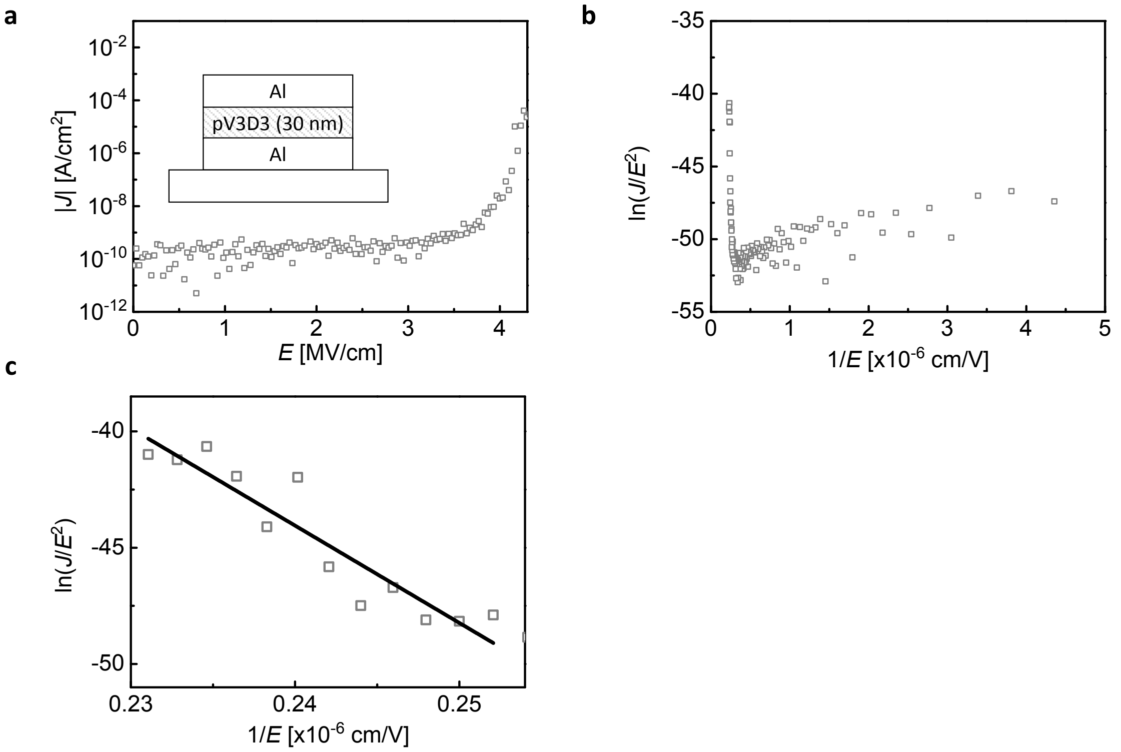


**Supplementary Figure S4. Conduction mechanism of pV3D3 insulating layer.** a) Measured *J-E* characteristic and b) its Fowler-Nordheim (F-N) plot of a metal-insulator-metal device with a 30 nm-thick pV3D3 insulating layer. c) is showing F-N plot in the region of F-N tunneling with fitted curve of the F-N tunneling equation. Tunneling based electric conduction was implied in the result of uniform *J-E* characteristics over wide range of temperature in the previous study[2]

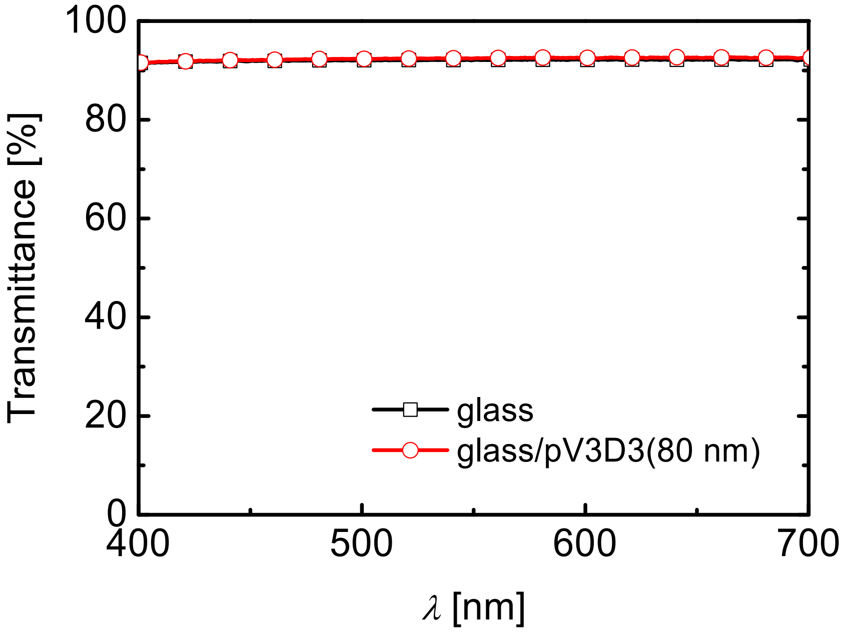


**Supplementary Figure S5. Transmittance of pV3D3.** Measured transmittance of a glass substrate and a 80nm-thick pV3D3 layer on the glass substrate.

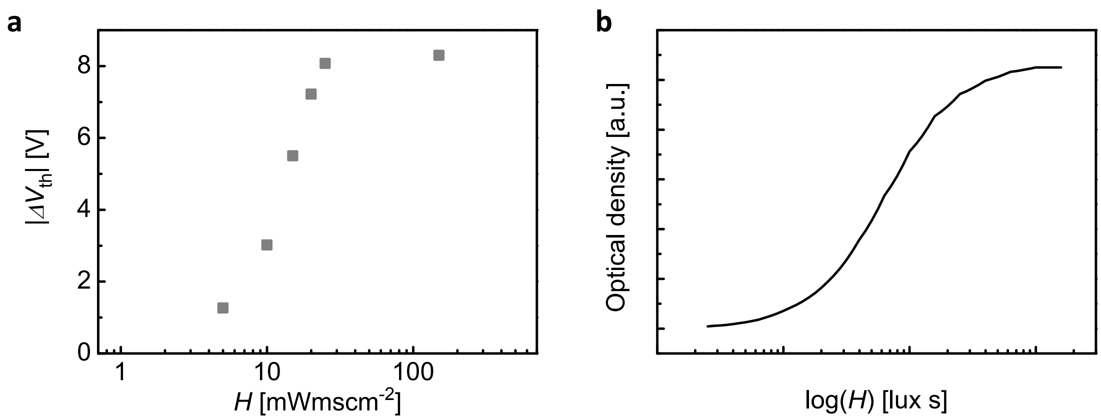


**Supplementary Figure S6. The light-program response of the proposed device upon the exposure.** a) Light-program response vs the exposure (*H*) which is redrawn from Fig. 2b and b) typical characteristic curve of photographic film. The overall measurement was performed in the region of normal exposure range as it is comparing to the characteristic curve of photographic film and the results showed the saturation at the high exposure as in the photographic film. This implies that the proposed OPMs have a proper range of operation with the same manners of photographic techniques in the practical implementation; a long exposure time for the dim light and a short exposure time for the bright light.

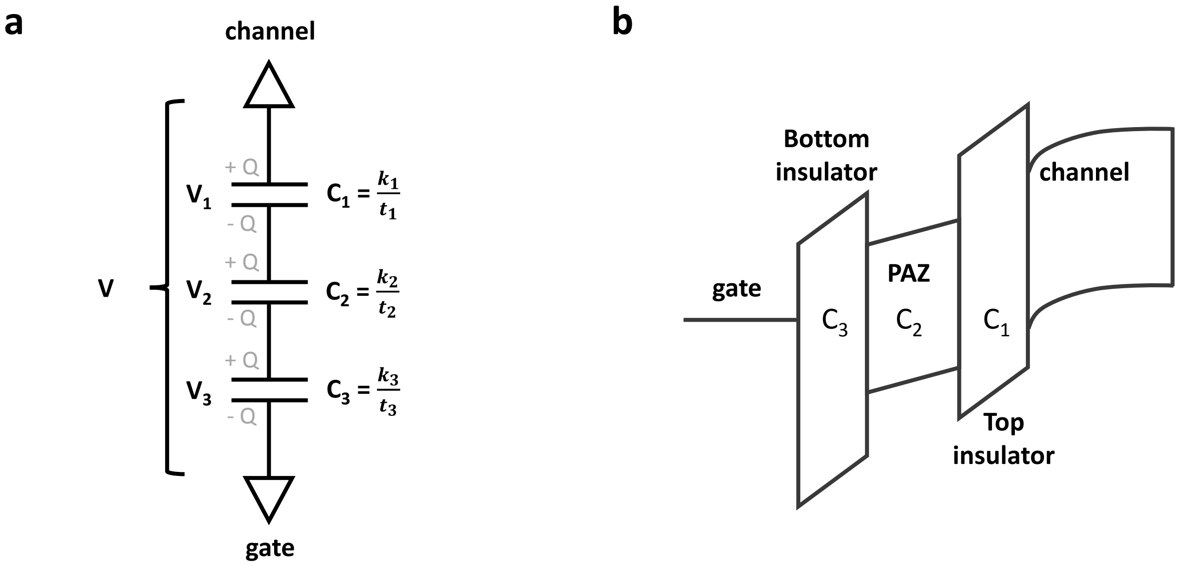


**Supplementary Figure S7. Equivalent capacitor model of the proposed device.** a) Equivalent capacitor model for calculating electric field induced by gate bias over each layer. b) Annotation of each capacitor in corresponding device structure. Each dielectric layers and PAZ regarded as equivalent capacitors with their corresponding dielectric constants and thicknesses. The dielectric constant of pV3D3, $k_1$ & $k_3$, and $C_{70}$, $k_2$ was considered as 2.2 and 4.0, respectively.

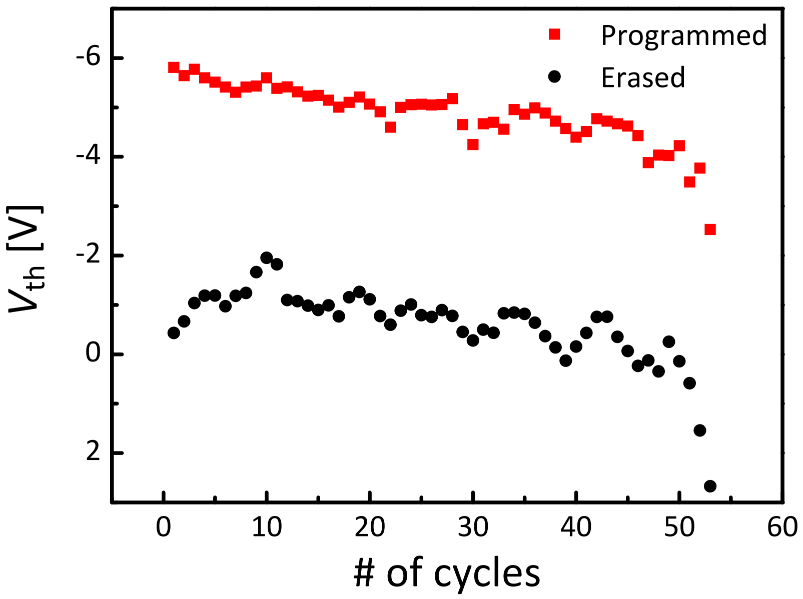


**Supplementary Figure S8. Endurance of the proposed device.** Endurance of the proposed device. The device in Fig. 4 was characterized for this endurance test. The device was photo-programmed with white LED (3.76 mWcm$^{-2}$) and 50 ms, 22 V of gate pulse and electrically erased by 200 ms, 40 V of gate pulse. The device was failed after 50 times of program-erase cycling because the abrupt increase of the gate leakage current. The tendency of slight decrease in both of programmed $V_{th}$ and erased $V_{th}$ is considered to be originated from the positive bias illumination stress during the photo-program.

## Supplementary Reference


1. Moon, H., Kim, M. & Yoo, S. Bilayer Source/Drain Electrodes Self-Aligned With Fluoropolymer Dielectrics for Stable High-Performance Organic TFTs. IEEE Electron Device Lett. 32, 1137-1139 (2011).

2. Moon *et al.*, Synthesis of ultrathin polymer insulating layers by initiated chemical vapour deposition for low-power soft electronics. *Nat Mater* **14**, 628-635 (2015)
